# A randomized, phase II study of the anti-insulin-like growth factor receptor type 1 (IGF-1R) monoclonal antibody robatumumab (SCH 717454) in patients with advanced colorectal cancer

**DOI:** 10.1002/cam4.263

**Published:** 2014-06-06

**Authors:** Edward H Lin, Heinz-Josef Lenz, Mansoor N Saleh, Mary J Mackenzie, James A Knost, Kumudu Pathiraja, Ronald B Langdon, Siu-Long Yao, Brian D Lu

**Affiliations:** 1 Seattle Cancer Care Alliance, University of WashingtonSeattle, Washington; 2 USC Norris Comprehensive Cancer CenterLos Angeles, California; 3 Georgia Cancer SpecialistsAtlanta, Georgia; 4 London Health Sciences CentreLondon, Ontario, Canada; 5 Illinois Cancer CarePeoria, Illinois; 6 Merck & Co., Inc.Whitehouse Station, New Jersey

**Keywords:** Colorectal cancer, IGF-1R, monoclonal antibody, robatumumab

## Abstract

Overexpression of insulin-like growth factor receptor type 1 (IGF-1R) may promote tumor development and progression in some cancer patients. Our objective was to assess tumor uptake of fluorodeoxyglucose by positron-emission tomography in patients with chemotherapy-refractory colorectal cancer treated with an anti-insulin-like growth factor receptor type 1 (anti-IGF-1R) monoclonal antibody, robatumumab. This was a randomized, open-label study with two periods (P1 and P2). Patients were randomized 3:1 into treatment arms R/R and C/R that received, respectively, one cycle of 0.3 mg/kg robatumumab or one or more cycles of second-line chemotherapy in P1, followed in either case by 10 mg/kg robatumumab biweekly in P2. The primary measure of fluorodeoxyglucose uptake was maximum standardized uptake value (SUV_max_). The primary endpoint was the proportion of patients in the R/R arm having a mean percent decrease from baseline in SUV_max_ (DiSUV) greater than 20% 12–14 days postdose in P2. Secondary endpoints included Response Evaluation Criteria in Solid Tumors (RECIST)-defined tumor response and pharmacodynamic measures of target engagement. Among 41 patients who were evaluable for the primary endpoint, seven (17%, 95% CI 7%–32%) had DiSUV greater than 20%. Fifty robatumumab-treated patients were evaluable for RECIST-defined tumor response and six (12%) had stable disease lasting greater than or equal to 7 weeks in P2. Pharmacodynamic endpoints indicated target engagement after dosing with 10 mg/kg robatumumab, but not 0.3 mg/kg. The most frequently reported adverse events were fatigue/asthenia, nausea, anorexia, and gastrointestinal disturbances. In this study, few patients with chemotherapy-refractory colorectal cancer appeared to benefit from treatment with the IGF-1R antagonist robatumumab.

## Introduction

Signaling by insulin-like growth factors 1 and 2 (IGF-1 and IGF-2) and their cognate receptor IGFR is important in normal ontological development, adult physiology, and in the development and progression of many cancers 1,2. Coexpression of IGFR and epidermal growth factor receptor is a prognostic factor in cancer of the lung 3, head, and neck 4, and in colorectal cancer 5, and it has been implicated in resistance to therapy with gefitinib 6. In an immunohistochemical analysis of tumors from 713 patients, Peters et al. found that 7.5% stained positive for IGF-1, 12.6% for IGF-2, and 99.6% for IGFR type 1 (IGF-1R) 7. An association has been hypothesized between overexpression of IGF-1R and patient survival, but this remains controversial 8–10. Overexpression of IGF-2 has been observed in normal liver tissue adjacent to metastases of colorectal cancer and such overexpression correlates positively with the proliferative index in such tumors 11,12. In an epidemiology study, it was found that higher levels of circulating IGF-1 were associated with greater future risk for colorectal cancer and higher levels of IGF-binding protein 3 (IGFBP-3) were associated with decreased risk 13. Similarly, lower pre-diagnosis plasma concentrations of IGF-binding protein 1 (IGFBP-1) have been linked to increased mortality in patients with colorectal cancer treated by surgical resection 14.

There is thus considerable interest in the development of agents that disrupt IGFR-mediated signaling for use as antitumor agents, and some of these have produced evidence suggestive of antitumor efficacy in phase I clinical trials 1,15. In phase II–III trials, anti-IGF-1R agents, as monotherapy and in combinations with other agents, have produced mixed results 2,16–18.

Robatumumab (also known as 19D12 and SCH 717454) is a fully human anti-IGF-1R monoclonal antibody of the immunoglobulin G1 (IgG1)/kappa isotype. It binds to the extracellular portion of human IGF-1R selectively and with high affinity, and thereby prevents IGF binding and activation of transduction events, including IGF-1R autophosphorylation, insulin receptor substrate 1 phosphorylation, and activation of downstream intracellular signaling events 19. Robatumumab has been shown to inhibit tumor growth in various human tumor xenograft models, to induce IGF-1R degradation, and to induce killing of tumor cells through the mechanism of antibody-dependent cellular cytotoxicity 19,20. In a phase I clinical study (unpubl.), treatment with robatumumab in doses from 0.3 to 20 mg/kg elicited substantial effects on pharmacodynamic markers including profound reductions in the numbers of IGF-1R-positive peripheral blood mononuclear cells (PBMCs) identified by fluorescence-activated cell sorting (FACS) analysis, large increases in serum IGF-1 and IGFBP-3, and effects on serum IGF-2 and IGF-binding protein 2 (IGFBP-2).

Positron-emission tomography (PET) has been widely used to assess changes in tumor uptake of 18 F-fluorodeoxyglucose (FDG). Uptake of FDG correlates with glucose metabolism in tumors, and changes in FDG uptake may be predictive for treatment effects on cancer cell proliferation and clinical endpoints in the treatment of colorectal cancer 21–23. The primary goal of this study was to assess whether treatment with robatumumab alters FDG uptake in patients with chemotherapy-refractory metastatic colorectal cancer. Additional goals included evaluation of the safety and tolerability of robatumumab in such patients, evaluation of treatment responses by Response Evaluation Criteria in Solid Tumors (RECIST), and measurement of pharmacodynamic serum biomarkers to assess target engagement.

## Patients and Methods

This was a randomized, fixed-sequence, multisite, open-label phase II study of robatumumab in adult patients with chemotherapy-refractory colorectal cancer. It was performed at 11 sites in the United States and Canada between 05 February 2008 and 04 June 2009. This study was registered with ClinicalTrials.gov (NCT00551213) and conducted in full accord with the principles of Good Clinical Practice. The study protocol was approved by appropriate institutional review boards and regulatory agencies, and patients provided written informed consent prior to their participation.

### Patients

Eligible patients were older than 18 years of age and had histologically confirmed colorectal carcinoma that had progressed during treatment with at least one form of first-line therapy. They had at least one index lesion that was greater than or equal to 3 cm in diameter and had a tumor-to-background ratio greater than or equal to 2:1 when analyzed for standardized uptake value (SUV) by FDG-PET. Eligible patients had an Eastern Cooperative Oncology Group (ECOG) performance status less than or equal to 2 and a life expectancy greater than or equal to 4 months.

### Study procedures

**Figure 1:**
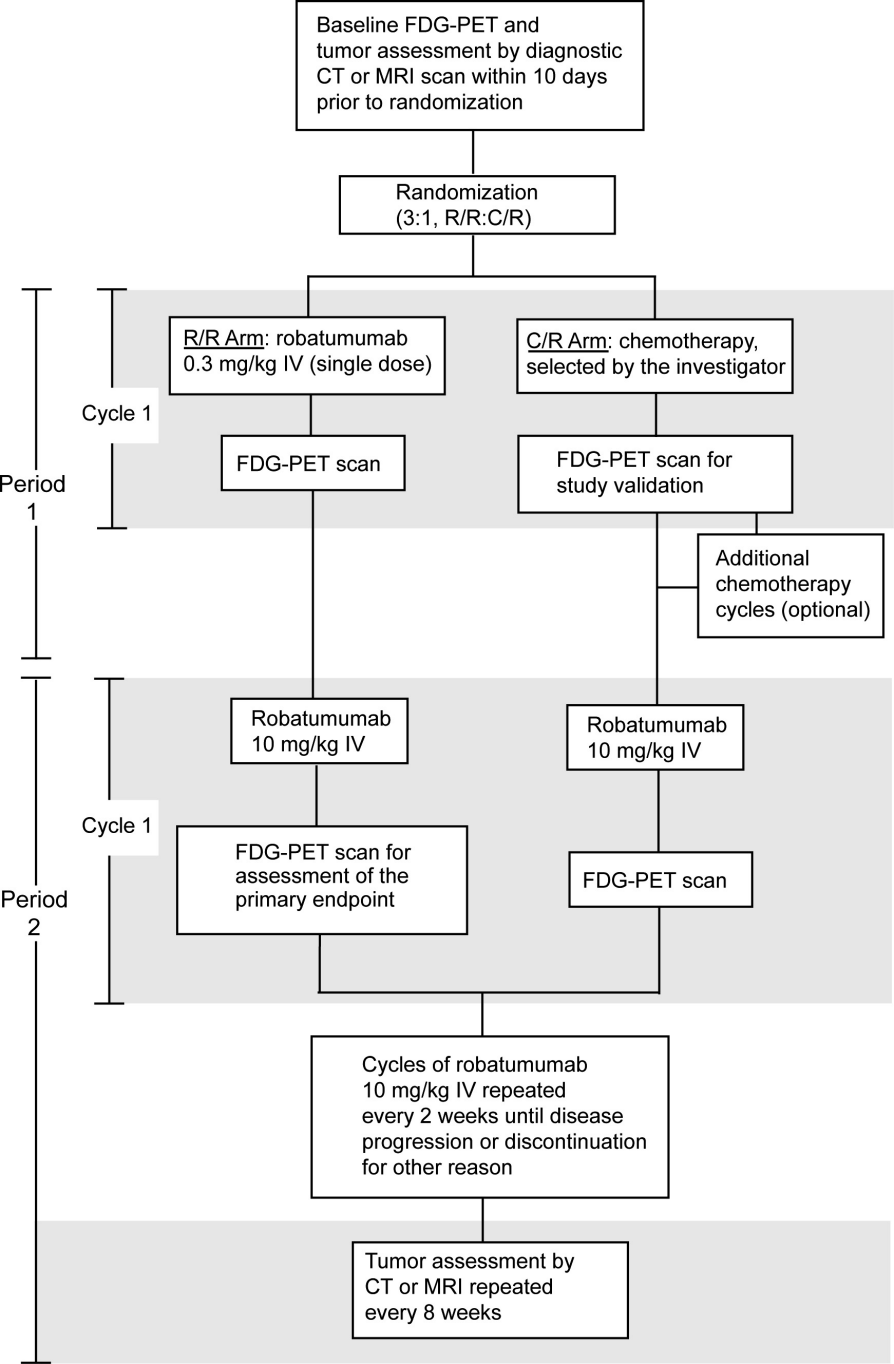
Study design.

Screening was followed within 28 days by randomization (3:1) into an robatumumab/robatumumab (R/R) treatment arm in which patients received robatumumab in two periods (P1 and P2) and a chemotherapy/robatumumab (C/R) treatment arm in which they received chemotherapy in P1 and robatumumab in P2 (Fig.1). Maximum standardized uptake value (SUV_max_) was used as the primary measure of FDG uptake into tumors. Baseline assessments of tumors by FDG-PET and magnetic resonance imaging (MRI) or X-ray computed tomography (CT) were performed within 10 days of randomization. On treatment, mean percent decrease from baseline in SUV_max_ (DiSUV) was calculated for each patient and timepoint by averaging the percent decreases observed for all target tumors.

The C/R treatment arm was included in the study to provide confirmation (in patients who had not yet received robatumumab) that the study design and execution adequately enabled observation of FDG-PET responses. It was prespecified that randomization of patients into the C/R arm would end once DiSUV greater than 20% was observed in P1 in three patients in that arm. The specific chemotherapy that C/R patients received in P1 was left to the investigator's discretion; patients remained on such treatment for as many cycles as were considered medically appropriate.

Patients in the R/R arm were treated with one cycle (one dose) of 0.3 mg/kg robatumumab in P1 and then entered P2, in which all patients received 10 mg/kg robatumumab. Treatments in P2 were begun 14 days after last treatments in P1 and were repeated every 2 weeks until disease progression. Robatumumab doses were delivered intravenously via a 60-min infusion into the forearm or a central venous catheter.

The first on-treatment FDG-PET images were acquired at the end of P1/C1, 12–14 days after dosing. A second FDG-PET scan was performed at the end of the first cycle in P2 (P2/C1) and this was prespecified as the primary timepoint for evaluation of robatumumab's effect on FDG uptake. Tumors were assessed by MRI or CT every 8 weeks in P2 and evaluated by RECIST 1.1 24.

Blood samples were obtained throughout the study for analysis of pharmacodynamic markers, anti-robatumumab antibodies, and circulating tumor cells (CTCs).

Safety was assessed by monitoring adverse events (AEs), electrocardiogram (ECG) parameters, laboratory tests, vital signs, and CD4 levels. Patients were also evaluated in a post study visit 4–5 weeks after the last dose of robatumumab.

### Endpoints

The primary endpoint was the proportion of patients in the R/R arm who had DiSUV greater than 20% (a “metabolic response”) at the end of P2/C1. Secondary and exploratory endpoints included RECIST-defined tumor response rate, duration of tumor responses, counts of IGF-1R-positive PBMCs, serum concentrations of IGF-I, IGF-II, IGFBP2, and IGFBP3, and counts of CTCs. Findings of stable disease (SD) were based on images acquired after at least 7 weeks of treatment in P2. Duration of response was defined as the combined durations of intervals of complete response (CR) and partial response (PR).

### Image analysis

All FDG-PET, MRI, and CT scans were de-identified and transferred in digital form to a contract research organization (RadPharm [now CoreLab Partners], Princeton, NJ) for independent, blinded analysis.

### Statistical methods

The primary analysis included all patients in the R/R arm with an evaluable DiSUV at the end of P2/C1. The percentage of these patients with DiSUV greater than 20% and its 95% confidence interval (CI) were calculated. For the analysis of change in pharmacodynamic serum biomarkers, mean percent changes and standard errors (SEs) were calculated. The sample size was planned to provide greater than or equal to 80% power to detect a 25% metabolic response rate in the R/R arm (compared with an expected background response rate of 10%) using a Chi-square test with an overall significance level of 0.05 (two-tailed).

The analysis of robatumumab immunogenicity included all patients whose serum tested negative for anti-robatumumab antibody at baseline and for whom there was at least one measurement of serum anti-robatumumab antibody concentration after treatment with 10 mg/kg robatumumab in P2.

Adverse events that began in P1 and P2 were summarized separately and AEs were tabulated by treatment.

## Results

### Patient disposition

**Figure 2:**
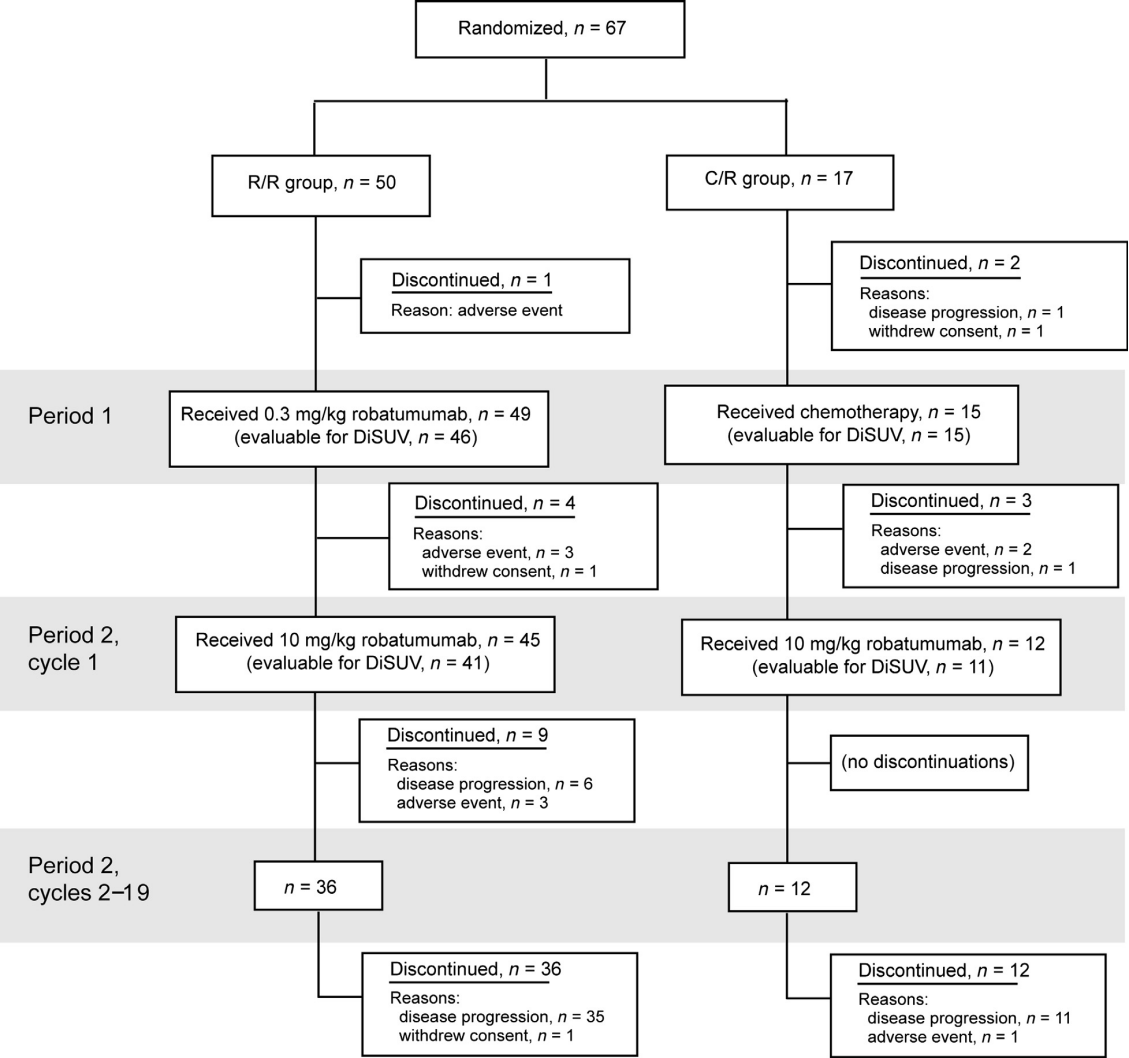
Patient disposition.

Sixty-seven patients were randomized overall: 50 into the R/R treatment arm and 17 into the C/R arm. Baseline demographic and disease characteristics were generally similar in these two treatment arms (Table1). Sixty-one of these patients received at least one dose of robatumumab and 57 received at least one dose of 10 mg/kg robatumumab (Fig.2).

**Table 1:** Patient demographic and disease characteristics at baseline.

Characteristic	Chemotherapy/robatumumab group (*n* = 15)	Robatumumab/robatumumab group (*n* = 49)	All patients (*n* = 64)
Gender, n (%)			
Male	6 (40)	19 (39)	25 (39)
Female	9 (60)	30 (61)	39 (61)
Age, years			
Mean (SD)	61.1 (11.0)	64.7 (10.9)	63.8 (10.9)
Median (range)	63.0	66.0	64.5
Racial origin, n (%)			
White	12 (80)	40 (82)	52 (81)
Black or African American	1 (7)	5 (10)	6 (9)
Multiracial	1 (7)	2 (4)	3 (5)
Asian	0	2 (4)	2 (3)
Pacific Islander	1 (7)	0	1 (2)
Ethnicity, n (%)			
Hispanic or Latino	0	2 (4)	2 (3)
Non-Hispanic or Latino	15 (100)	47 (96)	62 (97)
ECOG Performance status, n (%)			
0	5 (33)	21 (43)	26 (40)
1	8 (53)	27 (55)	35 (55)
2	2 (13)	1 (2)	3 (5)
Number of prior oncology therapies			
Mean (SD)	3.9 (2.2)	4.5 (2.6)	4.4 (2.5)
Median (range)	4 (1–9)	4 (1–13)	4 (1–13)

SD, standard deviation.

Patients in the C/R arm received 1–17 cycles of chemotherapy in P1 before entering P2. Patients who entered P2 remained there for up to 19 cycles of treatment. The mean durations of treatment in P2 were 3.8 and 4.1 cycles (∼8 weeks) in the R/R and C/R groups, respectively. All patients eventually discontinued, usually because of progressive disease (PD). Twenty-eight deaths were reported overall, including one prior to receipt of any study drug, six after treatment in P1 without entry into P2 (three in each treatment arm), and 19 in P2.

### Changes in FDG uptake

**Figure 3:**
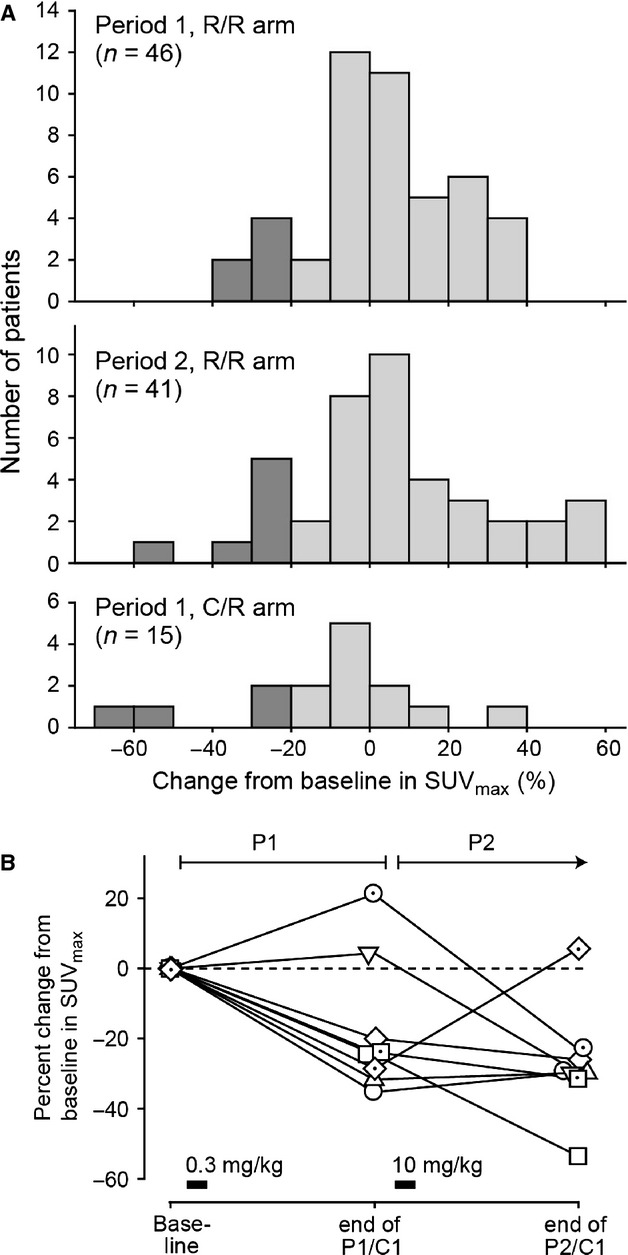
Distributions of change from baseline in mean SUV_max_ (A) in patients in the R/R group in P1 and P2, and in patients in the C/R treatment arm in P1 after their first cycle of chemotherapy. The threshold for positive metabolic response was predefined as decrease in mean SUV_max_ (DiSUV) greater than 20% (dark shading). (B) The timecourse of changes in mean SUV_max_ in the subset of patients in the R/R group who had DiSUV greater than 20% either in P1 or 2, or both. Horizontal bars indicate timing and doses of robatumumab.

DiSUV was evaluable in 46 and 41 patients in the R/R treatment group at the end of P1/C1 and P2/C1, respectively (Fig.3A). In P1/C1, DiSUV varied between 35% and −37% with a mean of −4.1% (i.e., 4.1% increase from baseline in SUV_max_); six patients met the prespecified metabolic response criterion of DiSUV greater than 20%. In P2/C2, DiSUV varied between 54% and −57% with a mean of −5.0%; seven patients had DiSUV greater than 20%. Thus, the percentage of patients meeting the prespecified criterion for metabolic response in P2 was 17% (95% CI, 7% to 32%).

In the C/R group, 15 patients had an evaluable DiSUV at the end of P1/C1 and values ranged from 69% to −36% (Fig.3). Four (27%) patients had DiSUV greater than 20% at the end of P1/C1.

There were three patients in whom DiSUV was substantially greater at P2/C1 than at P1/C1, and one patient in whom the converse was true (Fig.3B). Mean log-transformed SUV_max_ at P1/C1 and P2/C1 differed by 0.01 (95% CI, −0.08 to 0.05). Among the eight patients who had DiSUV greater than 20% following robatumumab treatment either in P1 or P2, six patients did so in P1 after having received only 0.3 mg/kg robatumumab.

### RECIST assessment of tumor responses

Among the 57 patients who entered into P2 and received at least one treatment with 10 mg/kg robatumumab, 50 were considered by central reviewers to be evaluable for RECIST-defined tumor response. Analysis by the central reviewers determined that 0 (0%) and six (12% of 50, 11% of 57) had tumor responses of PR and SD, respectively. As determined independently by the study's principal investigators, 54 of these 57 patients were evaluable for tumor response and one (2%) and six (11%), had PR and SD, respectively. The central reviewers’ and principal investigators’ evaluations differed for 19 of these 57 patients who entered P2. In three of these, it was a question of whether the patient response was evaluable for RECIST-defined response; in 15, it was whether the best response was SD and PD, and in 1, it was a question of whether the response was PR or SD. In this latter case, the patient was treated for 268 days (19 cycles in P2) without apparent disease progression.

Patients with SD generally had values for SUV_max_ in P2 within or below 20% of baseline. However, in none of the patients determined by central review to have had RECIST-defined SD was this outcome preceded by finding an FDG-PET-defined metabolic response (DiSUV greater than 20%) in P1 or P2.

### Pharmacodynamic effects and analysis of robatumumab immunogenicity

**Figure 4:**
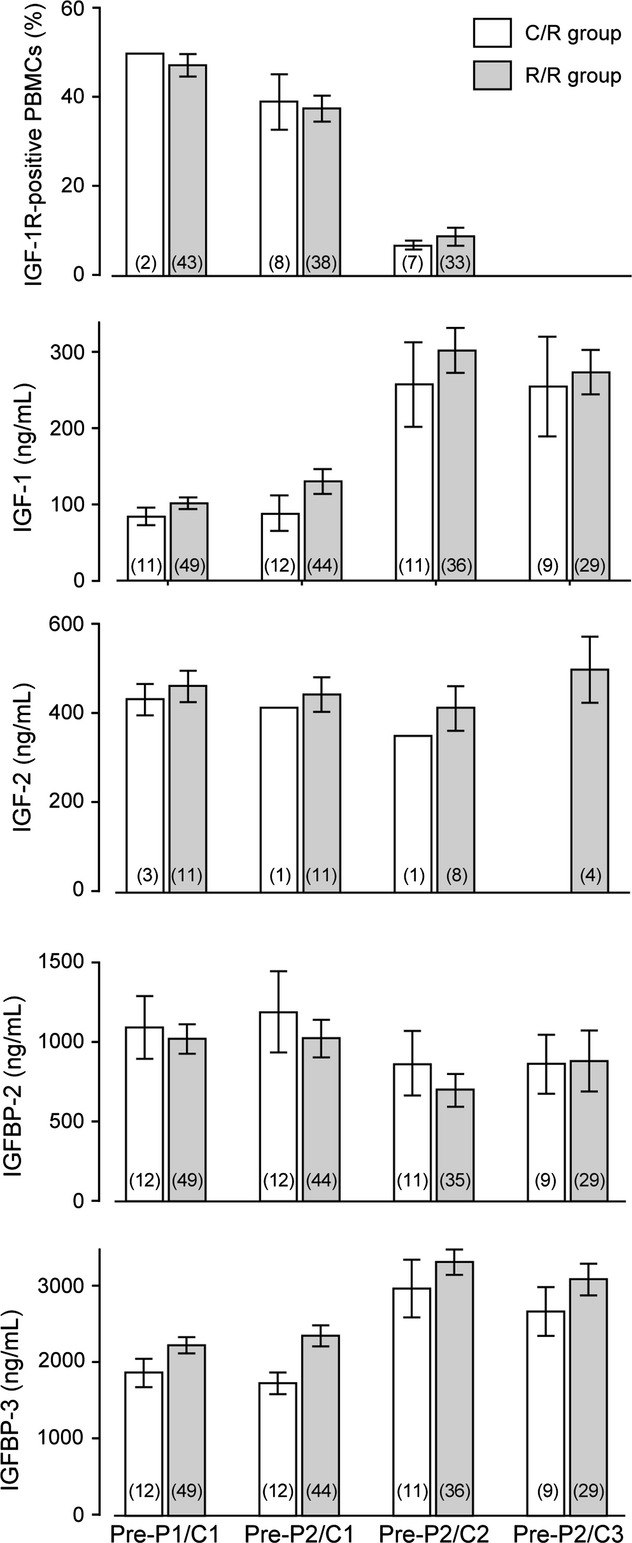
Serum concentrations of pharmacodynamic protein biomarkers and in percent of PBMC that were positive for surface IGF-1R prior to the first infusion of study drug (in P1/C1) and at posttreatment timepoints P2/C1, P2/C2, and P2/C3. Means ±SE are shown; the numbers of patients evaluable at each timepoint are indicated in parentheses.

Treatment with 10 mg/kg robatumumab in P2 was associated with an 80% reduction in counts of IGF-1R-positive PBMCs and two to threefold increases in mean serum IGF-1 concentration (Fig.4). Moderate decreases and increases were also observed in serum IGFBP-2 and IGFBP-3 concentrations. Serum IGF-2 appeared to be unaffected. No pharmacodynamic effects were observed until after patients had been treated with 10 mg/kg robatumumab in P2 (the pre-P2/C2 timepoint in Fig.4, assessed 12–14 days after the first treatment with 10 mg/kg robatumumab). Numerically, mean percentages of IGF-1R-positive PBMCs fell by 15%–20% between baseline (pre-P1/C1) and the first on-treatment measurement (pre-P2/C1), but this decline was observed in both the C/R group (which had received no robatumumab at that timepoint) and the R/R group.

The analysis of robatumumab immunogenicity included 42 patients who tested negative for anti-robatumumab antibody at baseline and were evaluable for anti-robatumumab antibody titer in P2. None of these patients had measurable levels of anti-robatumumab antibody posttreatment. Regardless of treatment status, very few CTCs were found in blood samples.

### Safety

Treatment-emergent adverse events (TEAEs) were reported in every patient. The most frequently reported TEAEs were fatigue, nausea, anorexia, diarrhea, vomiting, and abdominal pain (Table2). Primarily, they occurred after patients had received chemotherapy or 10 mg/kg robatumumab. Most TEAEs were of Common Terminology Criteria for Adverse Events (CTCAE) grade 1 or 2**.** Among TEAEs of grade 3/4 severity, the most frequently reported were abdominal pain (in 11% of patients in the R/R treatment group in P2) and fatigue (in 8% of C/R group patients in P2).

**Table 2:** Percent incidences of treatment-emergent adverse events (includes all specific events with incidence greater than or equal to 10% in any period in either treatment group).

	Period 1				Period 2			
Group:	C/R (*n* = 15)		R/R (*n* = 49)		C/R (*n* = 12)		R/R (*n* = 45)	
Grade:	Any	3/4	Any	3/4	Any	3/4	Any	3/4
Fatigue	33	0	4	0	42	8	20	2
Nausea	33	0	2	0	25	0	40	4
Anorexia	27	0	8	0	33	0	27	2
Diarrhea	33	7	6	0	33	0	29	2
Vomiting	20	0	6	0	17	0	29	4
Abdominal pain	27	7	6	4	0	0	27	11
Rash	27	0	0	0	0	0	2	0
Alopecia	7	0	0	0	25	0	7	0
Decreased appetite	0	0	2	0	25	0	2	0
Musculoskeletal pain	0	0	0	0	25	0	4	0
Constipation	20	0	12	2	25	0	13	2
Weight decreased	20	0	2	0	0	0	9	0
Dehydration	7	7	2	0	17	0	20	4
Cough	0	0	6	0	17	0	9	0
Back pain	0	0	0	0	17	0	7	2
Urinary tract infection	0	0	0	0	17	0	4	0
Dyspnea	13	0	0	0	8	0	4	0
Asthenia	13	0	2	0	8	0	13	0
Dry skin	13	0	2	0	8	0	2	0
Stomatitis	13	0	0	0	8	0	2	0
Gait disturbance	13	7	2	0	0	0	0	0
Headache	7	0	4	0	0	0	13	0
Hyperglycemia	0	0	0	0	8	0	13	2
Neuropathy peripheral	13	0	4	0	0	0	2	0
Dry mouth	0	0	0	0	0	0	11	0
Pain in extremity	0	0	0	0	0	0	11	2

## Discussion

In this FDG-PET study of responses to robatumumab in patients with chemotherapy-refractory colorectal cancer, 17% of patients treated with 10 mg/kg robatumumab subsequently met the prespecified FDG-PET response criterion of DiSUV greater than 20%. This rate of response fell short of the 25% that had been targeted. RECIST-defined SD was subsequently observed in 12% of evaluable patients. The intervals of SD were generally short-lived in this study and most patients were subsequently discontinued because of disease progression. In one patient, RECIST-defined SD lasted for approximately 9 months. This patient was a 70-year-old male who had previously received one chemotherapy regimen (fluorouracil, leucovorin, and oxaliplatin) beginning 195 days prior to study entry and ending 8 days prior to entry.

Target engagement was confirmed in this study by measuring counts of PBMCs that stained positive for IGF-1R. Although the underlying mechanism remains uncertain, it is known that robatumumab induces IGF-1R degradation 20 and profound decreases in blood counts of IGF-1R-positive PBMCs were observed in normal subjects in the first-in-human study of robatumumab (unpublished). In subjects administered 0.3 mg/kg robatumumab in that study, the percentage of PBMCs positive for IGF-1R fell to 9% of baseline between dosing and postdose day 8 and returned to 54% of baseline by postdose day 15. Treatment with this same dose had no apparent effect on this endpoint in this study, but given that IGF-1R-positive PBMCs were not measured until 14 days postdose, it is possible that this treatment produced a transient pharmacodynamic effect that went undetected. In any case, this study provided evidence for a strong pharmacodynamic effect in patients administered 10 mg/kg robatumumab.

As further evidence for target engagement, we observed numerical increases in mean serum IGF-1 and IGFBP3 in this study following treatments with 10 mg/kg robatumumab (but not after 0.3 mg/kg robatumumab). These effects were consistent with observations made previously in an unpublished phase I study of this agent and they are consistent with published mechanistic and epidemiological data 25–27.

Treatment with 10 mg/kg robatumumab in P2 did not generally elicit a greater DiSUV response than that elicited by treatment with 0.3 mg/kg robatumumab in P1. Thus, the FDG-PET findings in this study did not align well with the dose-dependency observed for effects on pharmacodynamic markers of target engagement.

Tumor resistance to robatumumab in this study may have been due in part to the fact that this was a heavily pretreated population of patients. All had tumors that had been unresponsive or had become refractory in prior treatments, with a median of four prior chemotherapies. Even so, the findings in this study suggest that robatumumab is likely to have limited value as a treatment for advanced colorectal cancer in general patient populations. Outcomes in recently completed Phase II and III studies with other anti-IGF-1R antibodies support a similar conclusion 16,28,29. Complexity in the IGF-1R signaling pathway may contribute tumor resistance to anti-IGF-1R agents; there are a large number of alternative signaling pathways that function in colorectal tumor biology 30.

Robatumumab appeared to be generally well tolerated in this study. Similar AE profiles have been reported in patients treated with the human anti-IGF-1R monoclonal antibody AMG479 15,31. Hyperglycemia is a known class effect of IGF-1R directed agents. Thrombocytopenia, transaminitis, and gastrointestinal AEs may also be mechanism-based toxicities.

In summary, the findings in this study and other recently completed studies suggest that monoclonal antibodies directed against IGF-1R may have little utility as a treatment for colorectal cancer in unselected patient populations. However, a subset of patients with refractory colorectal cancer may have transiently responded to robatumumab in this study. While dysfunction of the IGF pathway appears to be critically important in colorectal cancer carcinogenesis, much work is still needed to develop the best approach to integrating anti-IGFR therapy into the treatment of colorectal cancer.
